# Sir Victor Horsley

**Published:** 1916-08

**Authors:** 


**Sir Victor Horsley** died of heat stroke in Mesopotamia on July 16.

